# Vasopressor-Induced Digital Ischemia

**DOI:** 10.7759/cureus.16595

**Published:** 2021-07-23

**Authors:** Shruti Jesani, Sherif Elkattawy, Muhammad Atif Masood Noori, Sarah Ayad, Suha Abuaita, Kirolos Gergis, Omar Elkattawy, Vipin Garg

**Affiliations:** 1 Internal Medicine, Rutgers New Jersey Medical School/Trinitas Regional Medical Center, Elizabeth, USA; 2 Internal Medicine, St. George's University, True Blue, GRD; 3 Internal Medicine, McLaren Health Care, Flint, USA; 4 Internal Medicine, Rutgers New Jersey Medical Center, Newark, USA; 5 Internal Medicine, Pulmonology, Rutgers New Jersey Medical School/Trinitas Regional Medical Center, Elizabeth, USA

**Keywords:** digital ischemia, vasopressor, norepinephrine, neo-synephrine, vasopressin

## Abstract

In patients who are critically ill and in circulatory shock, substantial dosages of vasopressors including norepinephrine and Neosynephrine are often required to sustain blood pressure. While these medications are necessary and can be lifesaving, they are often associated with several complications related to severe vasoconstrictions. One of these known but underreported side effects is digital ischemia (DI). DI refers to a decrease in digital perfusion. It is a rare and uncommon phenomenon that can lead to significant consequences and unfortunately can result in amputation of the digits. Herein, we report an unfortunate female with septic shock secondary to acute bowel ischemia who developed bilateral digital necrosis while on norepinephrine.

## Introduction

Digital ischemia (DI) is described as the reduction in digital perfusion. It is an uncommon pathology yet severe enough that it generally leads to amputation. Given that the management of digital ischemia varies depending upon the etiology, it is vital to be familiar with differential diagnosis. It includes embolism, atherosclerosis, connective tissue diseases, drug use and occupational injury. Pre-existing peripheral vascular disease, concomitant use of vasopressors and prolonged hypotension can increase the risk of vasopressor-induced digital ischemia. Vasopressor therapy is one of the important causes of DI, especially in the intensive care unit. Critically ill patients with circulatory shock requiring high doses of vasopressors, especially norepinephrine, can be associated with poor outcome due to excessive vasoconstriction. We describe a patient with acute bowel ischemia and shock who developed bilateral digital necrosis while on norepinephrine, Neosynephrine and vasopressin. According to Adverse Drug Reaction Probability Scale (Naranjo Scale), a probable relationship between the adverse effect (digital necrosis) and vasopressor therapy in this patient exists.

## Case presentation

A 57-year-old female with past medical history of hypertension, diabetes mellitus, multiple sclerosis, and celiac disease presented to emergency department with abdominal pain secondary to chronic constipation. CT scan of the abdomen was significant for colonic obstruction secondary to fecal impaction in the rectosigmoid region with marked dilatation of the colon with cecum measuring up to 7 cm. Patient was being managed for fecal impaction with lactulose, magnesium citrate, psyllium and nasogastric tube was placed for decompression. Patient had multiple small bowel movements, but her abdomen remained rigid and distended without full resolution of fecal impaction. Patient's abdominal pain worsened and she was found to be hypotensive and tachycardia. Fluid bolus was given but patient remained hypotensive hence was intubated and upgraded to the ICU for further management. CT abdomen was remarkable for extensive pneumatosis and portal venous gas, highly concerning for ischemia, diffuse bowel dilatation with significant proximal small bowel dilatation, mild distal small bowel dilatation and diffuse colonic dilatation. Patient was treated with fluids and antibiotics and was started on norepinephrine to maintain systolic blood pressure and mean arterial pressure. Surgery was consulted and patient underwent total colectomy, ileostomy, gastrostomy but few patchy areas of necrotic small bowel were also noted hence patient underwent small-bowel resection and stricturoplasty of jejunum. Post-surgery, patient developed severe acute respiratory distress syndrome with severe hypoxemia. Chest X-ray was remarkable for bilateral infiltrates, more so on the right side.

**Figure 1:**
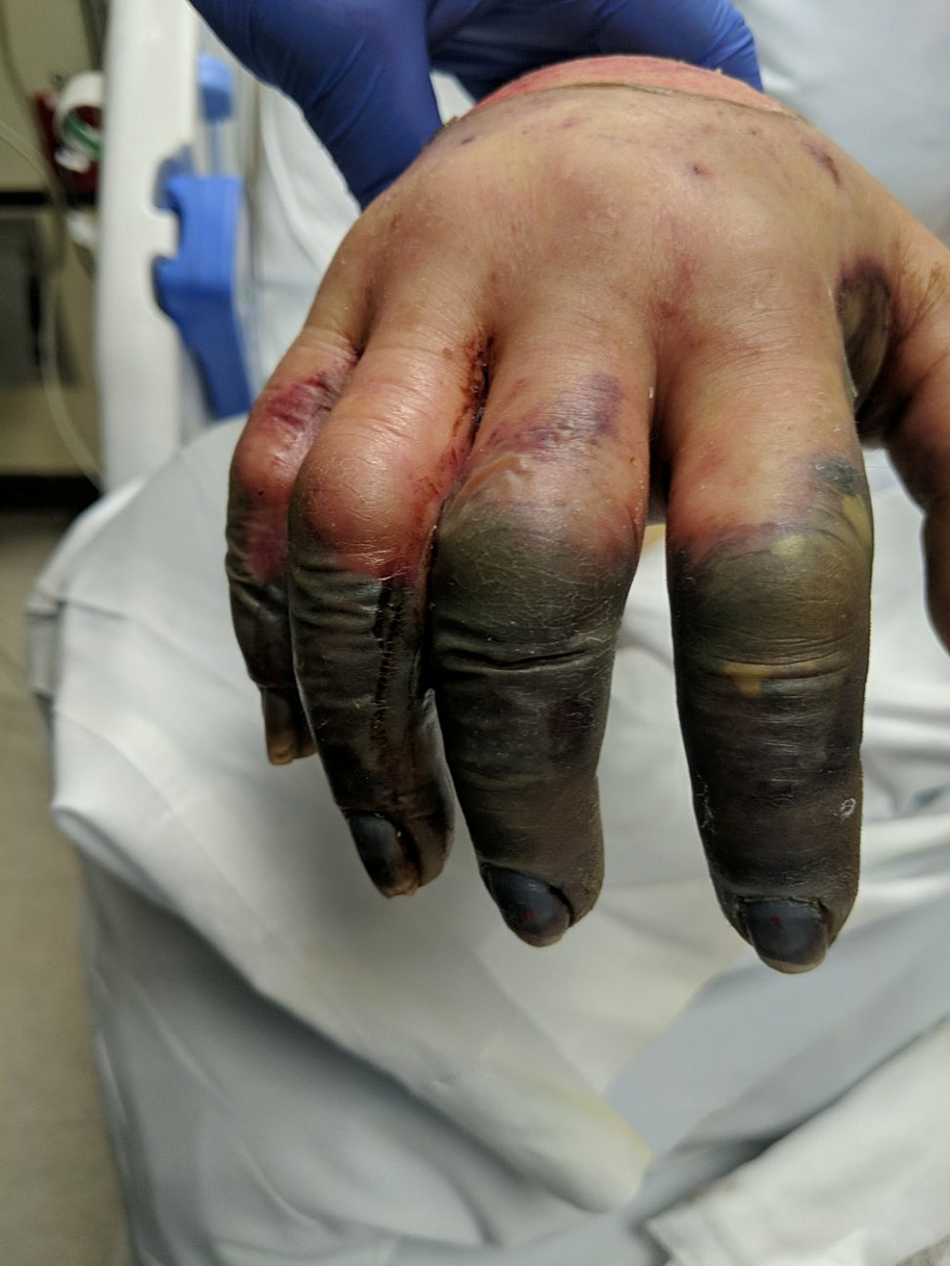
Right hand significant for digital ischemia

**Figure 2:**
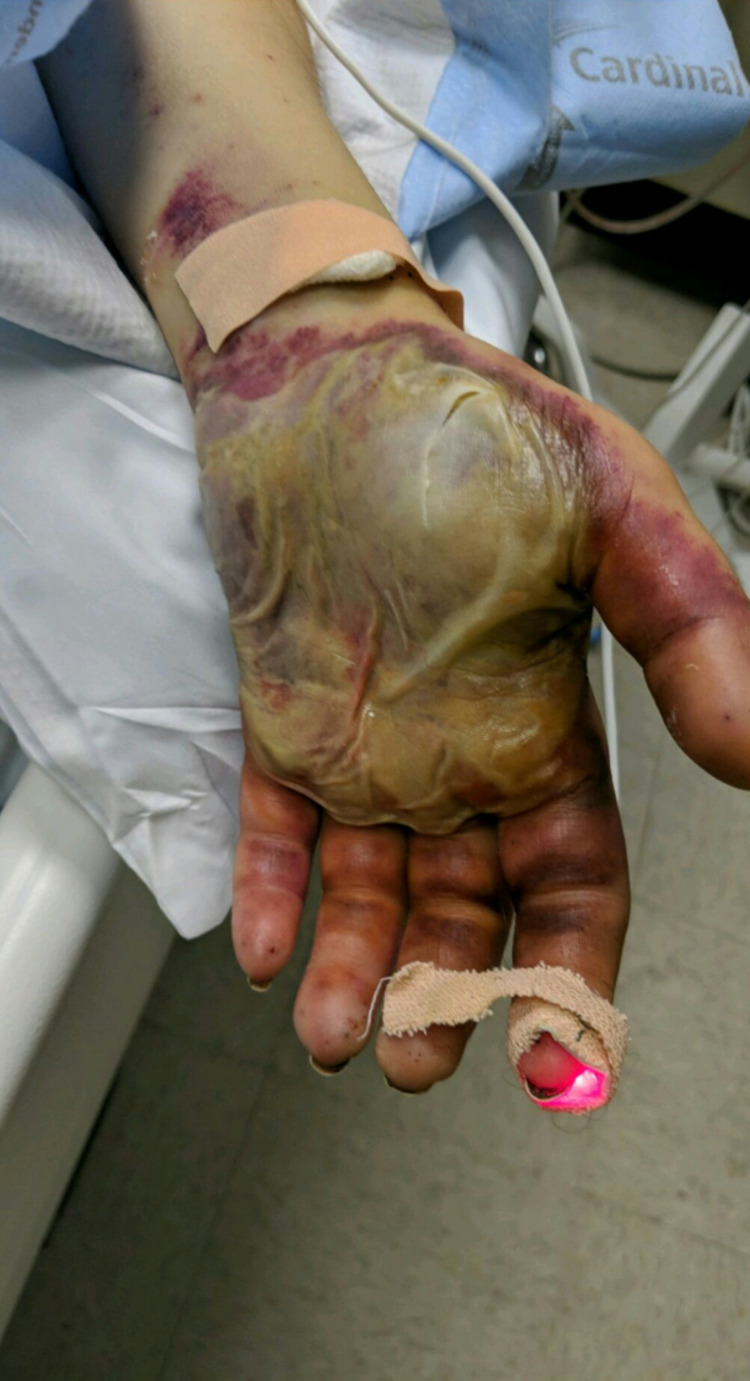
Left hand significant for digital ischemia and fluid filled blister

Patient’s condition remained critical and she was kept on the maxed dose of norepinephrine, phenylephrine and vasopressin. Patient’s renal function was deteriorating and she was started on dialysis daily to correct metabolic acidosis. Patient's liver enzymes were also trending upward secondary to hypoperfusion. Bluish discoloration of the right and left hand were noted with capillary refill more than three seconds. Radial pulses were dopplerable and the skin was intact and cool to touch. In three to four days, fluid-filled bullae with gangrenous changes were noted on both hands as seen in Figure 1 and Figure 2.

Patient's condition remained critical, and prognosis was poor; a family meeting was held and the decision was made for DNR/DNI. Ultimately they decided to terminally extubate patient and make her comfort care. 

## Discussion

Vasopressors, which are administered intravenously, cause vasoconstriction, or they could be used to increase contractility in patients with shock. Increasing vasoconstriction will ultimately lead to an increased systemic vascular resistance (SVR), causing an increase in mean arterial pressure (MAP) therefore increasing perfusion to organs. Vasopressors work on both alpha and beta receptors [1]. Alpha receptors are peripheral vasoconstrictors hence they cause the increase in SVR. Beta 1 receptors have positive chronotropic effect on the heart rate and positive inotropic effect on the contractility of the heart. On the other hand, beta 2 receptors work as vasodilators to the organs [1]. There is an increase in arterial pressure due to constriction of venous vessels that increases venous blood pressure thus increases preload and cardiac output.

Vasopressors are typically given for extremely ill patients such as ICU patients. In patients with shock, a high dose of vasopressors is needed in order to maintain blood pressure until antibiotics and volume repletion takes over [2]. In our case, vasopressors were used due to the fact that our ICU patient had shock due to ischemia of the small and large intestines. Our patient was given three vasopressors since shock was worsening. Our patient was given vasopressor support through a central line as needed for a goal of MAP > 65 mmHg. There are many types of vasopressors, such as dopamine, dobutamine, norepinephrine, epinephrine, isoproterenol, phenylephrine, etc [3]. Each drug functions differently based on its affinity to alpha versus beta receptors. Specifically, our patient was on norepinephrine, phenylephrine and vasopressin. Norepinephrine acts as a vasoconstrictor and is used in severe hypotensive emergencies [4]. Norepinephrine is a potent alpha 1 agonist making it a very powerful vasoconstrictor [4] and has some beta 1 receptor agonist activity resulting in positive inotropic effects on the heart at higher doses. Furthermore, vasopressors have multiple complications, some of which are arrhythmia due to beta stimulation, hypertension, reflux bradycardia, and peripheral digital ischemia. Our patient presented with digital ischemia after the administration of vasopressors.

Peripheral digital ischemia occurs due to inadequate blood supply to the digital tissue. Studies have shown the occlusion of small blood vessels when the intraluminal pressure falls below a critical value leads to compromised flow through digital arteries at perfusion pressures between 36 and 60 mm Hg. Due to vasoconstriction, there is less blood flow going to the extremities such as the digits and instead organs are more perfused. The first sign of digital tissue loss is when there is pain with pallor or cyanosis of the skin [5]. Our patient presented with digital gangrene on the proximal interphalangeal joints and distal interphalangeal joints of the right hand with mild ischemia on the left hand. Symmetrical digital gangrene has been linked to low cardiac output, disseminated intravascular coagulation, autoimmune conditions, and drugs such as norepinephrine and epinephrine [6]. The dosage of the vasopressor is determined by age, BMI, MAP, and the patient's cardiovascular condition. The dosage has been linked to causing digital ischemia even at recommended levels [6].

In our patient, when one vasopressor was started there were no signs of digital ischemia. As soon as three vasopressors were started due to worsening shock, digital ischemia was noted. In a specific study where high dose vasopressor therapy was given due to septic shock, digital or limb necrosis was noted in six patients. As our patient’s condition was worsening, her condition remained critical and prognosis was poor, patient was made DNR/DNI and ultimately extubated and made comfort care.

## Conclusions

Vasopressors are cornerstone therapy in the ICU. They are technically “life support for blood pressure.” However, they, indeed, come with a risk of digital ischemia and necrosis. Vasopressors with alpha adrenergic activity such as norepinephrine are more likely to sustain limb ischemia. These drugs should be used judiciously, and continuous efforts should be made to taper them off as soon as possible. Counteractive management should be started once discoloration of an extremity is detected, to salvage the extremity and avoid function loss. Collectively, vasopressors are the definition of scientific advancement, however like other lifesaving medications, they come with risks.
